# Interpretable deep learning for gastric cancer detection: a fusion of AI architectures and explainability analysis

**DOI:** 10.3389/fimmu.2025.1596085

**Published:** 2025-05-29

**Authors:** Junjie Ma, Fang Yang, Rong Yang, Yuan Li, Yongjing Chen

**Affiliations:** ^1^ Department of Gastrointestinal Surgery, Shanxi Province Cancer Hospital/Shanxi Hospital Affiliated to Cancer Hospital, Chinese Academy of Medical Sciences/Cancer Hospital Affiliated to Shanxi Medical University, Taiyuan, Shanxi, China; ^2^ Head and Neck Radiotherapy Ward, Shanxi Province Cancer Hospital/Shanxi Hospital Affiliated to Cancer Hospital, Chinese Academy of Medical Sciences/Cancer Hospital Affiliated to Shanxi Medical University, Taiyuan, Shanxi, China; ^3^ Department of Gastroenterology, Shanxi Province Cancer Hospital/Shanxi Hospital Affiliated to Cancer Hospital, Chinese Academy of Medical Sciences/Cancer Hospital Affiliated to Shanxi Medical University, Taiyuan, Shanxi, China

**Keywords:** gastric cancer, deep learning, explainable AI, explainable representations, convolutional neural networks, VGG16, RESNET50, MobileNetV2

## Abstract

**Introduction:**

The rise in cases of Gastric Cancer has increased in recent times and demands accurate and timely detection to improve patients' well-being. The traditional cancer detection techniques face issues of explainability and precision posing requirement of interpretable AI based Gastric Cancer detection system.

**Method:**

This work proposes a novel deep-learning (DL) fusion approach to detect gastric cancer by combining three DL architectures, namely Visual Geometry Group (VGG16), Residual Networks-50 (RESNET50), and MobileNetV2. The fusion of DL models leverages robust feature extraction and global contextual understanding that is best suited for image data to improve the accuracy of cancer detection systems. The proposed approach then employs the Explainable Artificial Intelligence (XAI) technique, namely Local Interpretable Model-Agnostic Explanations (LIME), to present insights and transparency through visualizations into the model's decision-making process. The visualizations by LIME help understand the specific image section that contributes to the model's decision, which may help in clinical applications.

**Results:**

Experimental results show an enhancement in accuracy by 7\% of the fusion model, achieving an accuracy of 97.8\% compared to the individual stand-alone models. The usage of LIME presents the critical regions in the Image leading to cancer detection.

**Discussion:**

The enhanced accuracy of Gastric Cancer detection offers high suitability in clinical applications The usage of LIME ensures trustworthiness and reliability in predictions made by the model by presenting the explanations of the decisions, making it useful for medical practitioners. This research contributes to developing an AI-driven, trustworthy cancer detection system that supports clinical decisions and improves patient outcomes.

## 1 Introduction

Gastric cancer (GC) has evolved as a widely spread deadly malignancy across the globe, resulting in increased death rates and severe implications due to factors like Helicobacter Pylori Infection, gastritis, Aging, Improper hygiene standards, etc. (1). Gastric cancer is one of the fifth most prevalent types of cancer diagnosed globally and stands out as the third most significant cause of death among cancer-infected patients (2, 3). Despite the advancements in medical and diagnostic facilities, the early detection of gastric cancer poses a critical challenge, primarily due to its vague and obscure symptoms (4). Traditional healthcare methods such as Biopsies and Medical Imaging are considered Invasive and have resource constraints (5). Early and accurate detection of GC is essential to improve survival rates and patient outcomes. More precise, non-invasive, reliable, and scalable solutions are needed to promote early diagnosis of Gastric Cancer (2).

Over the past years, Artificial Intelligence (AI) has revolutionized the domain of healthcare and medical diagnostics by assisting in enhanced decision-making. AI-driven models, especially the Deep Learning (DL) and the Machine Learning (ML) models, facilitate enhanced cancer detection by analyzing vast data volumes and recognizing complex patterns to detect the abnormalities that people may sometimes overlook (6). For Instance, Convolutional Neural Networks (CNN) analyze the endoscopic images to facilitate Image-based cancer detection. Subsequently, the Recurrent Neural Networks (RNN) and its variants, namely Long short-term memory (LSTM) and Bi-directional LSTM (BLSTM), have delivered promising results in analyzing sequential data as well as time series data such as genomic sequences (7, 8). Through automated lesion detection, segmentation, and classification made possible by integrating DL with real-time endoscopic operations, clinicians can now make choices more quickly and accurately (9).

Even though the individual DL models have shown remarkable success in particular tasks, combining several DL architectures has drawn many researchers’ attention to improve prediction performance (10). Fusion models have emerged as a promising way in AI-driven healthcare solutions, combining multiple DL modalities to enhance the performance of cancer detection systems (11). Other methods involving various modalities, like ensemble learning, stacking, and bagging, are also employed for detecting cancer (12). Model Fusion offers collaborative learning from different feature representations, integrated into a single model rather than combining the output of different models (13). Ensemble learning and stacking techniques face difficulties in cross-model interaction, which Intermediate Fusion offers. Fusion approach eliminates the need to maintain multiple complete models and tends to be computationally efficient compared to other techniques involving various modalities (14). A more thorough representation of the underlying data is extracted by leveraging the strengths of several models, such as CNN, VGG16, ResNet, EfficientB3, etc., and taking advantage of their distinct capacities for capturing the spatial representations from the Images (15). This study implements the intermediate fusion approach, where the individual models are first trained to extract intermediate representations or feature maps. These representations are concatenated, and the fused features are jointly trained before final prediction (13). This fusion approach provides individual model separation and combined collaborative learning. The intermediate fusion technique offers more benefits than early and late fusion. Early fusion tends to combine data from multiple sources, facing integration issues due to different data formats and statistical properties. Intermediate fusion allows the selection of the best-suited model for various data sources, avoiding the integration of raw data. Late fusion tends to combine the output of different modalities, limiting the cross-model interactions mitigated by the intermediate fusion technique (16). Intermediate fusion support joint training that allows the models to adapt to other models and learn complex patterns across different modalities, which is impossible in early and late fusion (17). The detection of Gastric cancer may become more accurate, resilient, and generalizable due to the intermediate-level model fusion. The comparison of different fusion approaches is presented in **Table 1**. However, combining several DL models adds more complexity, especially regarding the transparency and explanations of the models’ decisions (18).

**Table 1:** Comparison of different fusion approaches.

Criteria	Early Fusion	Late Fusion	Intermediate Fusion
Handling Data Heterogeneity	NO	YES	YES
Enabling Cross Model Interactions	NO	NO	YES
Supporting Joint Optimization	NO	NO	YES
Robustness towards missing data in one modality?	NO	NO	YES
Scalability	NO	NO	YES

The “black-box” character of DL models is one of its most significant drawbacks, which restricts their use in diagnostic contexts (19, 20). The traditional AI-driven models do not provide the relevant information and insights into the decisions made. Hence, it becomes difficult for clinicians to accept and validate the predictions made by them. For clinicians and other medical professionals to trust and successfully use AI tools in decision-making, they need systems that are interpretable and explainable (21). Explainable AI (XAI) has become the new domain for investigation to overcome this challenge (22). By emphasizing the essential aspects and decision processes, XAI techniques like attention mechanisms, saliency maps, and feature attribution shed information on how models make their predictions (23). XAI techniques evaluate the model predictions, find biologically significant patterns, and ensure the system’s outputs align with clinical knowledge in the context of GC diagnosis (24). This study implements LINE (Local Interpretable Model-Agnostic Explanations), which highlights the critical regions of the data, presenting an easy and clear explanation of the model’s decision (25). LIME is model-agnostic and works without knowledge of the internal model structure, which makes it applicable to various data sources. Other XAI methods like SHAP and GRADCAM are resource-intensive and are harder to interpret (26). In contrast to such methods, LIME is a lightweight technique and presents faster execution, which is necessary for critical applications like cancer detection.

**Figure 1:**
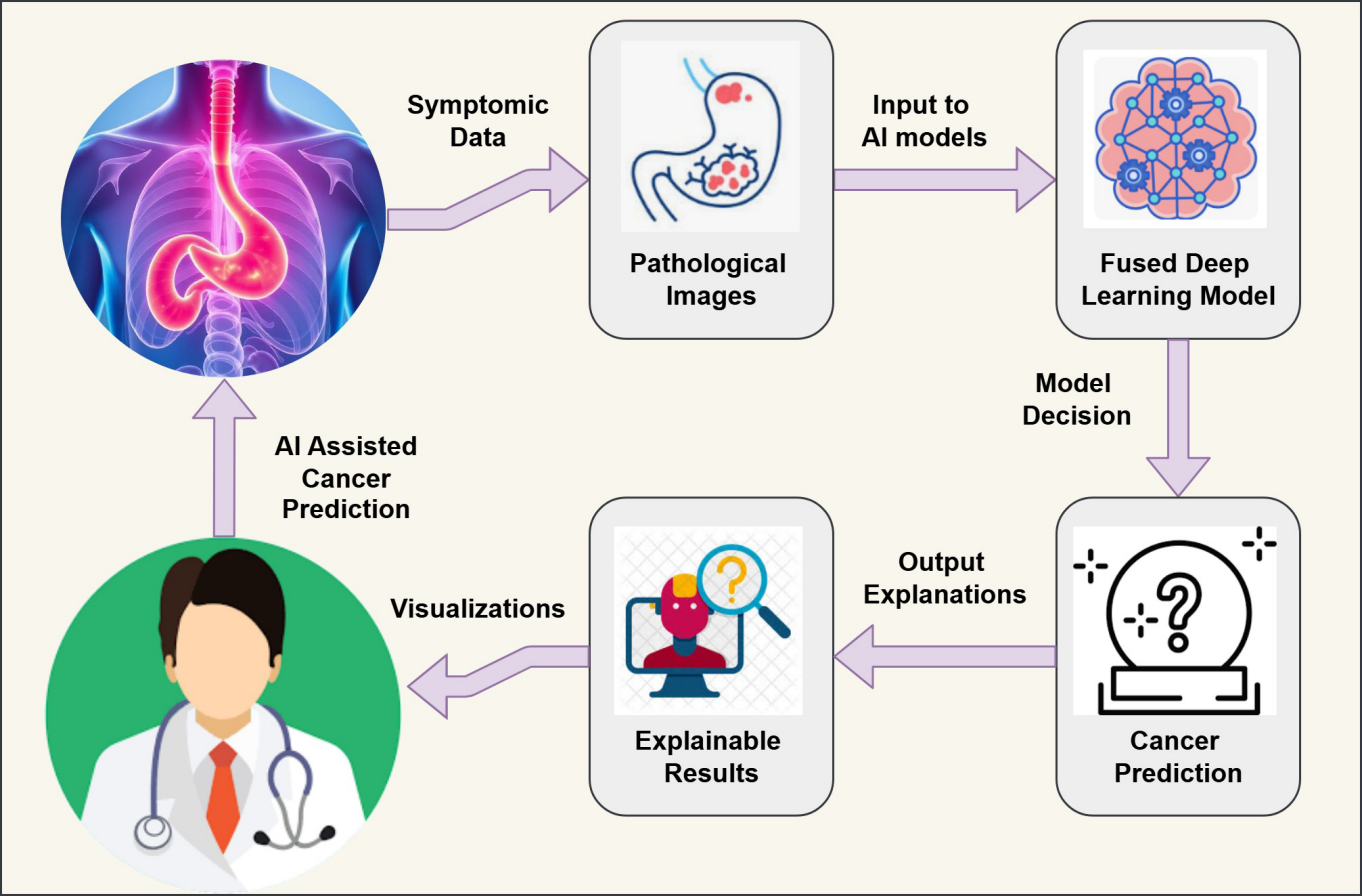
AI-assisted gastric cancer detection.

The primary motivation for our study arises from the need for the early & precise detection of GC to improve the survival rate. Even while AI-driven diagnostic systems have advanced, current models sometimes lack stability and interpretability, which reduces their suitability for clinical application (24). Additionally, gastric cancer cells’ complex and varied nature poses difficulties in analysis by the single-model approaches (6). This study intends to improve predicted accuracy while guaranteeing the explainability of AI-driven judgments by combining VGG16, RESNET50, and MobileNetV2 models. Applying the LIME technique gives physicians and clinicians visualizations and insights into how AI models make predictions. LIME presents the explanations at the local level by highlighting the areas in the images that contribute to the model’s decision. XAI promotes more well-informed decision-making and increases confidence in AI-assisted diagnostics by offering analytical and visual explanations of model outputs (27). AI-assisted cancer diagnostic systems assist healthcare professionals with well-informed, data-driven decisions. Our research aims to create a more reliable and interpretable AI system for gastric cancer detection, as demonstrated in **Figure 1**.

This study introduces a novel model fusion approach for gastric cancer detection with interpretability of results. The proposed framework leverages the strengths of three best-suited deep learning architectures through intermediate fusion and presents visual insights for the predictions through the XAI technique. It combines three deep learning models—VGG16, RESNET50, and MobileNetV2—to capitalize on their complementary feature extraction capabilities to improve classification accuracy and robustness. Furthermore, we use the XAI approach, LIME, to overcome the black-box nature of DL modalities, providing interpretable information about the decisions predicted by the model. Our proposed methodology achieves two primary goals: (1) A combination of multiple DL architectures to enhance the performance of the fusion model, and (2) implementation of the XAI technique to present the explanations about the model’s predictions to ensure transparency and a feeling of trust to the medical professionals. Through the integration of a fused DL model with explainability through XAI, we tend to fill in the gap between traditional clinical practice and AI-driven diagnostics, providing a way for a reliable gastric detection system. The major contributions of this paper are as follows.

- Design and Development of a unique framework for detecting gastric cancer by fusing the VGG16, RESNET50, and MobileNetV2.
- Application of XAI technique LIME for providing insights and visualizing the features contributing to model predictions.
- Performance Evaluation of the proposed methodology on the GasHisSDB dataset and demonstrating its superiority by comparing its performance with other approaches.

This article’s formation is as follows. Section 2 presents an overview of various methodologies designed and employed for Cancer detection. Section 3 presents the proposed method, describing all the relevant and minute details. Section 4 presents the experimental setup and varied parameters used in the experiments. The experimental results of our proposed methodology and a comparative study to evaluate the model’s performance compared to state-of-the-art approaches are presented in Section 5. Section 6 covers the Conclusion and the future scope.

## 2 Related work

Many researchers have developed varied and diverse methodologies for detecting Gastric Cancer. A systematic literature review is conducted to gain relevant insights into the existing literature in this domain, consisting of studies published over the last decade. Relevant and highly cited publications were selected from various platforms, including online databases and offline resources from academic libraries. In the first phase, searches were performed using keywords such as gastric cancer detection, medical imaging for gastric cancer, endoscopic analysis, DL and ML in cancer diagnosis, histopathological image analysis, and early detection strategies. Several well-established online databases were explored to compile relevant research, including IEEE Xplore, Springer, Elsevier, ACM Digital Library, Google Scholar, Scopus, arXiv, and PubMed. The second phase of the study involved defining inclusion and exclusion criteria. Studies focusing on gastric cancer detection through non-invasive techniques were prioritized, while research incorporating machine learning and deep learning methodologies with explanations was particularly emphasized. Only peer-reviewed articles were considered for analysis, whereas non-peer-reviewed studies, white papers, and research solely addressing preventive measures without diagnostic applications were excluded. In the final stage, articles were selected based on relevance, citation impact, reference quality, and indexing in reputed databases. This structured review approach helps identify key challenges and research gaps in gastric cancer detection.

A long time ago, the detection of Gastric cancer was solely dependent on the wisdom and expertise of radiologists and pathologists (28). The capability to learn and analyze data enables Artificial Intelligence (AI) to contribute significantly to medical image processing. AI Assistance in analyzing biomedical images and records has delivered promising results over the past years (29). The traditional ML models depend on handcrafted features determined by the experts of the domain (30). With the advancement in computational facilities and data availability in large volumes, deep learning is a powerful and promising tool in medical imaging. It has delivered promising results in detecting gastric cancers at an early stage (31, 32). The primary problem with deep learning models, their black-box nature, is eliminated with the advent of the XAI techniques that explain how the model derives the decision (33). Convolutional Neural Networks (CNN) are used in analyzing medical images and have recently increased (34). In (35), the researchers designed a CNN-based analysis of endoscopic images for detecting Early-Stage Gastric Cancer (EGC). This model was trained on a dataset of 13584 images comprising 2693 images of cancer lesions. This model presents a lower specificity, resulting in more false positives. The study in (36) introduced a CNN-based cancer diagnostic system employing the Inception-V3 model for detecting EGC. In this approach, the researchers train the CNN model using approximately 1700 images of EGC and nearly 400 images with non-cancerous lesions. The inception model works well in visualizing complex patterns and has recorded an accuracy of approximately 90%. The authors in (37) designed the CNN-based cancer detection system by analyzing endoscopic images using the RESNET50 model, known for its robust feature extraction. The training set consisted of around 800 images, and nearly 200 were used to test the model. This model achieved an accuracy of 89%, but the data volume used for training is low; for larger datasets, the results may decline. In (38), a CNN-based detection of gastric cancer from endoscopic images is demonstrated. The model is trained on nearly 13000 images and presents a sensitivity rate of 92%. The model achieved a fast processing speed, making it suitable for real-time clinical applications. This model observed a high false positive rate. Using single standalone models for detecting GC poses limitations in extracting enhanced features and results in low performance.

The research in (39) shows the ensemble learning mechanism’s use to overcome the individual models’ limitations. This approach combined the predictive power of RESNET34, RESNET50, and the VGG networks for detecting GC. This model results in higher computational times, limiting its application in real-world clinical applications. The study in (10) presents the ID-GCS (Intelligent Decision-Making for Gastric Cancer Screening), a multi-modal AI-driven diagnostic system for detecting cancer by integrating images with Textual data. The proposed model employs the hybrid attention mechanism to combine the spatial features from images and the textual semantics from endoscopic reports. The authors in (40) present a deep learning fusion model using deep feature extraction and optimization from the Wireless Capsule Endoscopy (WSE) images. This approach fuses the Inception-V3 and the DenseNet-201 models. The features extracted by individual models are concatenated using parallel concatenation, resulting in higher accuracy with higher computational times. The study in (41) presents a deep learning-based multi-modal feature fusion model designed to enhance the survival prediction of gastric cancer patients by integrating histopathological images, clinical data, and genomic information. This approach employs the GLFUnet and the Graph Convolutional Neural Network to extract gene expression data and features with enhanced representation of pathology images. In (42), a multi-scale feature fusion approach is presented that integrates the multi-scale features, resulting in improved detection of small and multiple lesions. With the help of encoders, better feature representations are facilitated. The authors in (43) propose GCLDNet, a novel deep learning framework for gastric cancer lesion detection that integrates multiple advanced techniques to enhance diagnostic accuracy. This approach implements the level feature aggregate structure and the attentionbased feature fusion module. This approach records the accuracy of 88% and 89% respectively, on the SEED and the BOT datasets consisting of histopathological images. Haq et al. in (44) designed the hybrid DL approach for multi-class classification of gastric cancer from endoscopic images. The proposed model integrates GoogleNet and Vision Transformers to classify Images into typical, early-stage, and advanced-stage cancer. The use of DL models causes a hurdle for clinicians to accept the results because of the unavailability of explanations about the decisions derived from the model.

The researchers in (19) present the use of XAI techniques to overcome the black-box character of the DL algorithms. This approach presents the use of Gradient-based models and shape-based feature extraction techniques on the outputs generated by RESNET50, AlexNet, and GoogleNet models. Additionally, LIME is applied to highlight the most critical regions influencing predictions, enhancing trust in AI-generated results. In (45), the researchers employed ensemble learning integrating InceptionV2 and V3, VGG16 models, and the explanations are presented by SHAP. The SHAP presents the feature-level interpretations of the model decisions, making it acceptable to clinicians. Shaw et al. in (46) demonstrate using the DenseNet-201 model for predicting cancer and explaining the results with LIME. With LIME, enhanced visualizations and validations of decisions are presented by highlighting the influential regions in the Images. The authors in (47) demonstrated using LIME to interpret the textual data from the patient’s health records. Various machine learning techniques analyze the data, and LIME interprets the results. In (21), SHAP is applied to interpret the feature importance based on the detection presented by hybrid deep learning architectures.

Advancements in deep learning, ensemble learning models, and XAI techniques for gastric cancer detection are reflected in the literature review. Various methodologies for GC have employed different CNN-based architectures, presenting enhanced feature extraction and improved classification results. When tested on diverse datasets, most standalone models encounter difficulties with low generalization, high false positive rates, and small lesion detection. Although ensemble models improve accuracy, their use in real-time clinical situations is hampered by their high computing costs and longer inference times. Moreover, while XAI techniques such as LIME, SHAP, GRADCAM, etc. have been introduced to address the black-box character of the DL algorithms. The SHAP and GRADCAM demand high computational resources and result in higher latency. Various approaches are implemented on limited datasets, limiting their ability to generalize effectively to real-world scenarios. Our proposed approach combines VGG16, RESNET50, and MobileNetV2 to address the limitations noted in the literature, effectively extracting multi-scale features while maintaining efficiency. LIME-based explanations are provided to present transparency, lower latency, and ensure the clinicians trust the AI-driven predictions. By optimizing feature fusion and balancing computational efficiency, our model aims to provide a more robust, interpretable, and clinically viable solution for gastric cancer detection.

## 3 Proposed methodology

**Figure 2:**
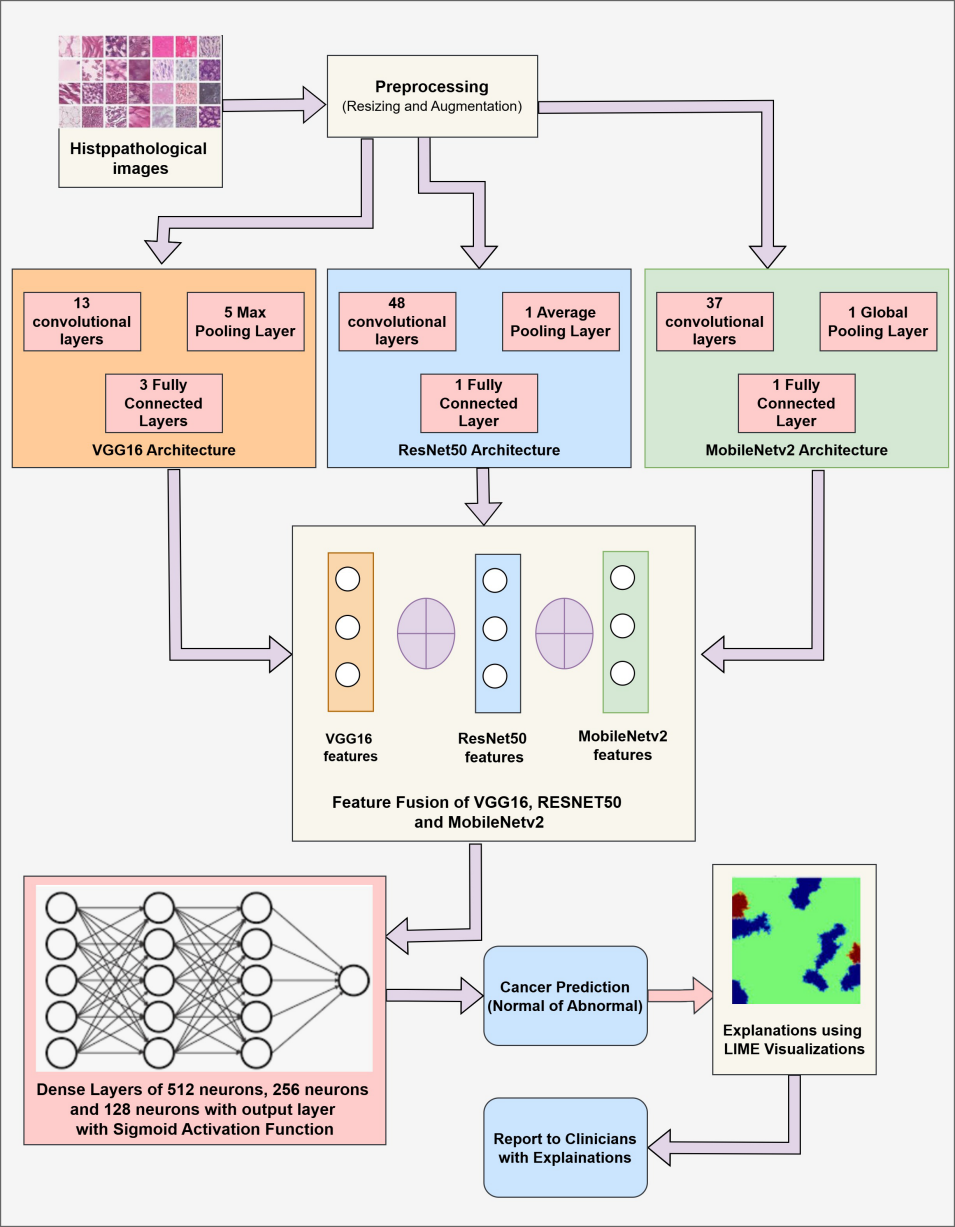
Architecture of fusion model with LIME interpretation.

The proposed methodology combines three CNN variant models, namely VGG16, RESNET50, and MobileNetV2. The primary goal of this fusion is to combine the strengths of each model and improve the overall feature extraction process. After prediction, LIME is applied to provide insights about the model’s decision, ensuring the transparency and trustworthiness of the model. The proposed methodology includes two components: (1) Cancer Prediction using a Fused Model by analysis of Images, and (2) Explaining the model’s decision using LIME visualizations. **Figure 2** presents the fused model’s architecture with relevant details.

Our proposed work integrates the three convolutional-based neural networks: VGG16, RESNET50, and MobileNetV2. The VGG16 model is a 16-layer model comprising 13 convolutional and 4 fully connected layers. This model is chosen because of its simplicity and capacity to capture minute details through small convolution filters (48). RESNET50 model is a 50-layer CNN network that employs residual connections to overcome the vanishing gradient problem by skipping some connections, resulting in more efficient learning (49). MobileNetV2 is a lightweight architecture consisting of depth-wise convolutions that can be separated and an inverted residual structure that reduces the computational complexities while maintaining the model’s accuracy (50).

The fusion is performed at an intermediate level. The outputs of the penultimate layers of each model are combined to achieve a comprehensive and enhanced feature representation. Each of the models captures different patterns from the data through partial model training. The patterns observed by these models are then concatenated to present a rich and distinct set of features and representations. A high-dimensional fused feature vector then undergoes further training through the fully connected dense layers. This fully connected layer incorporates the learned representations forwarded to the output layer. Later, the fully connected layer is followed by the output layer that uses the sigmoid activation function, which works well for binary classification.

LIME (Local Interpretable Model-agnostic Explanations) technique highlights the critical regions within the Image that contribute to the fused model’s decision-making process. LIME produces locally faithful explanations by altering the input image and tracking the alterations in the model’s predictions. Then LIME identifies and highlights the critical areas within the Image, and the non-important areas are shaded with black pixels. These visualizations by LIME help medical practitioners validate the model’s decision and build trust in the model’s decision-making process.

### 3.1 Proposed algorithm

The proposed algorithm comprises three popular CNN modalities: VGG16, RESNET50, and MobileNetV2. These individual models are implemented to fetch the spatial features from the Image data. These diverse features are fused at the intermediate layers, presenting more comprehensive feature vectors and leveraging the capabilities of individual networks. These fused vectors are then passed through a fully connected layer for final classification. Additionally, LIME is applied to present the visuals of the highlighted content in the Images that contribute towards the Model’s prediction for Gastric Cancer. The detailed algorithm for Gastric Cancer Detection is given in **Algorithm 1**.

As shown in **Algorithm 1**, the input is the set of the Histopathological Images D_I_ consisting of pairs (*x_i_,y_i_*) where *x_i_* represents the image and the *y_i_* represents the corresponding label (0 or 1). These images are resized to 160*160 pixels and are normalized by dividing them by 255 such that the data falls in the range of [0,1]. The input normalized data is supplied to the pre-trained CNN models, namely VGG16, RESNET50, and MobileNetV2. The VGG16 model comprises 13 convolutional layers, five pooling layers, and three fully connected layers. This model is computationally expensive but extracts the low-level features from the data, making it useful in medical imaging. The RESNET50 model consists of 48 convolutional layers, a single pooling layer, and a single fully connected layer. This model targets the vanishing gradient problems common in deep neural networks. This model is highly suited for extracting deep hierarchical features while being computationally efficient. MobileNetV2 is a lightweight deep neural network that presents depth-wise separable convolutions and inverted residual blocks that reduce the computational cost while preserving critical spatial features. The last layer, i.e., the fully connected layer of every individual model, is frozen, and the intermediate outputs representing features are obtained with the help of Global Average Pooling (GAP). This technique compresses the high-dimensional feature maps into low-dimensional vectors, resulting in lower complexity and retaining spatial information. These extracted features from individual modalities are concatenated, forming a comprehensive feature map. This fused feature map is then passed through three fully connected dense layers comprising 512 neurons, 256 neurons, and 128 neurons to learn complex patterns among the features. These layers employ the ReLU activation function to introduce non-linearity. A drop-out rate of 50% is applied to reduce the over-fitting of the model. Then, the output layer is employed with a sigmoid activation function to facilitate binary classification with either (0:Cancer Detection or 1:Cancer Not Detected) output. LIME is used to improve the interpretability of the model’s conclusions. Important areas of an image that affect the model’s predictions are found and highlighted by LIME. It operates by changing the input image and tracking the changes in the model’s output. Black pixels conceal non-relevant areas, while the key regions contributing to the classification choice are highlighted.

Algorithm 1 **Feature fusion of VGG16, RESNET50 and MobileNetV2 for gastric cancer classification.**

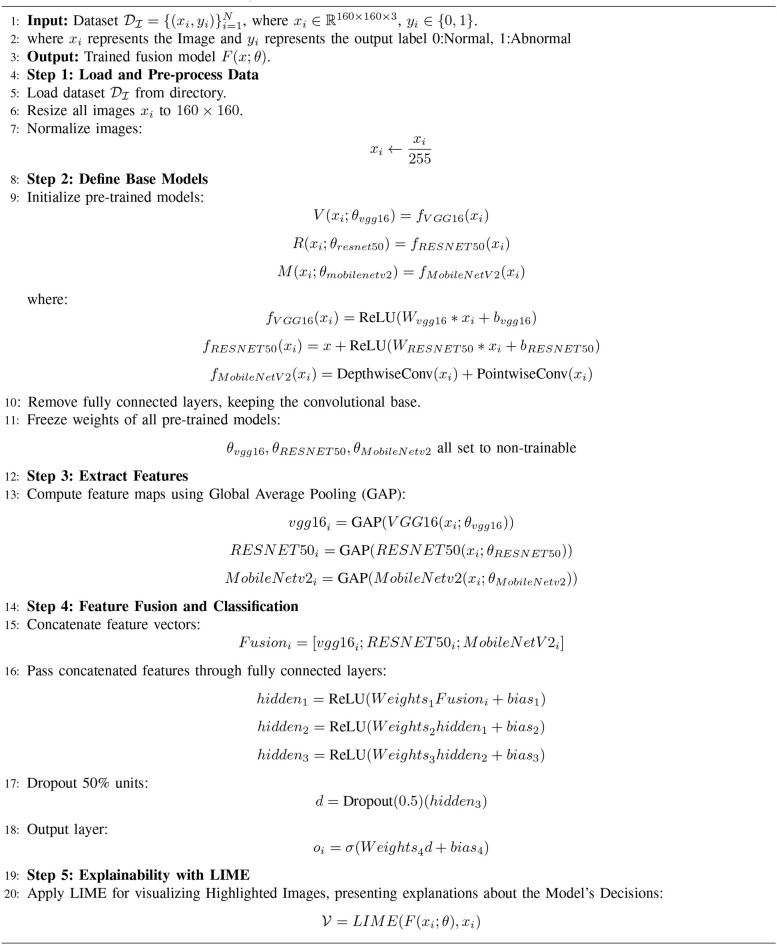

### 3.2 Complexity analysis

The computational complexity of the model is analyzed through the number of operations, the number of trainable parameters, and the time incurred at each step in the decision-making process. The time complexity includes the time spent in extracting the features by the individual model, fusing these features, and applying the LIME technique. The total complexity of the model is the summation of all these complexities incurred at different stages.

*1) Feature Extraction*: Each model extracts the features from the input image xi through numerous convolutional operations. The time complexity for a single convolution operation in any model is given by:


$$O(kernel^{2}\times C_{input}\times C_{output}\times Ht\times Wd)$$

Where:

- *kernal* represents the size of kernel (e.g., *kernal* = 3 for 3x3 kernels),
- *C_input_* and *C_output_* denote the input and output channels.
- *Ht* and *Wd* represent the height and width of the image.

Then, the Global Average Pooling is performed, which has complexity as shown below.


O(Ht×Wd)

The total feature extraction time for each model is denoted as:


$$T_{VGG16}=O\left(T_{VGG16}\right)$$

$$T_{ResNet50}=O\left(T_{ResNet50}\right)$$

$$T_{MobileNetV2}=O\left(T_{MobileNetV2}\right)$$

Where TVGG16, TResNet50, and TMobileNetV2 are the total time complexities for feature extraction of each model. The feature extraction process of each of these three models is performed in parallel, so the overall complexity for feature extraction is:


$$T_{extraction}=\text{max }(T_{VGG16},T_{ResNet50},T_{MobileNetV2})$$

*2) Feature Fusion*: The extracted features are concatenated to form a single high-dimensional feature map. The size of the fused vector is the sum of the dimensionalities of each model’s output. Let dimVGG16, dimResNet50, and dimMobileNetV2 represent the dimensionalities of the feature vectors for VGG16, RESNET50, and MobileNetV2, respectively. dimFusion represents the dimensions of the fused vector. The time complexity of the fusion process is denoted as:


$$\begin{array}{c} T_{Fusion}=O\left(dim_{Fusion}\right)\\ =O\left(dim_{VGG16}+dim_{ResNet50}+dim_{MobileNetV2}\right) \end{array}$$

*3) Fully Connected Layer:* The combined features are then passed through the fully connected layer. The time complexity of each of the dense layers is represented by:


$$O\left(dim_{input}\times dim_{output}\right)$$

where *dim_input_* represent the input dimensions and *dim_output_* denote the output dimensions. The total time complexity of dense layers will be


$$\begin{array}{c} T_{dense_{l}ayers}=O\left(dim_{fusion}\times hidden_{1}\right)+O\left(hidden_{1}\times hidden_{2}\right)\\ +O\left(hidden_{2}\times hidden_{3}\right)+O\left(hidden_{3}\times 1\right) \end{array}$$

Where *hidden*_1_, *hidden*_2_, and *hidden*_3_ denote the neuron counts at each hidden layer. The output layer consists of one neuron for binary classification.

*4) LIME Explainability:* LIME applies a perturbation operation to the input image to explain the model’s decision. Let *count* represent the number of perturbations. The time complexity of LIME is defined as:


$$T_{LIME}=O\left(count\times T_{Fusion}\right)$$

*5) Overall Complexity:* The total time complexity per image, including all the operations, is defined as:


$$T_{Overall}=T_{extraction}+T_{dense_{l}ayers}+T_{LIME}$$

Where *T_extraction_* is the total complexity for feature extraction, *T_denselayers_* is the complexity of the fully connected layers, and *T_LIME_* is the complexity of LIME explainability.

## 4 Experimental setup

Varied experiments are carried out to evaluate the performance of a fused deep learning architecture on the GasHisSDB dataset, specifically on the 160 * 160 pixels Image dataset. These experiments are carried out on an Intel Core i7 processor with NVIDIA GeForce 1650–4 GB GPU and 8 GB DDR4 RAM. The experiments are implemented using Python 3.7 within the Anaconda Framework. The DL models and XAI techniques were implemented using TensorFlow and Keras, and the LIME libraries explain the model. Additional Libraries, namely Numpy, SMOTE, and Matplotlib, were used for data handling and visualization.

The images are pre-processed by normalizing them to a standard scale to ensure data consistency. Image rotation and flipping are applied as a part of the augmentation process to create a generalized behavior in the model. The complete dataset is split into a ratio of 75:25, where 75% data is used for training, and 25% is used for evaluating the model. 10% of the training data is used for validation of the model. The images of size 160*160 pixels with three channels are fed to the fused model comprising three pre-trained CNN models (RESNET50, VGG16, and MobileNetV2). The model undergoes training for 50 epochs with a batch size of 32. The Regularization techniques, namely drop-out and batch normalization, help to improve the model’s consistency and prevent over-fitting.

The evaluation of the fused model is facilitated through different performance metrics, such as Accuracy, Precision, Recall, and F1 score. The XAI technique, LIME, is applied to interpret the decisions taken by the model. The application of LIME highlights the specific areas within the individual images that contribute to the model’s decision. Using XAI techniques ensures that clinicians understand the patterns observed by deep learning models are trustworthy and can be accepted.

### 4.1 Dataset description

The dataset used for the experiments is the Gastric Histopathology Sub-size Image Dataset (GasHisSDB), consisting of pathology images representing gastric cancer (51). This dataset incorporated 600 whole photos of 2048 * 2048 pixels, which were captured with a 20X magnification camera, and were annotated by four experienced pathologists at Longhua Hospital in China. TO generate more granular data, some researchers from Northeastern University extracted 245196 image patches, which were further investigated and validated by experienced pathologists from Liaoning Cancer Hospital and Institute. These image patches were captured at three distinct sizes: 80*80 pixels, 120*120 pixels, and 160*160 pixels, forming three subdatasets. These sub-datasets comprise two classes: Abnormal, denoting the presence of cancer tissues, and Normal, indicating the absence of cancer cells. In the case of Abnormal images, the image regions in the photos were carefully extracted, and the areas with a minimum of 50% cancerous tissue were retained to ensure high-quality data. The images are rotated to reduce the correlation among the image patches, and the whole dataset is scrambled to facilitate balanced distribution.

The Whole images were strained using the hematoxylin and eosin (H&E), a standard technique for straining Histopathology Images (52). These images were analyzed using high-precision microscopes to ensure robust cancer tissue visualization. This straining technique provides the best data for deep-learning architectures to detect gastric cancers. This study uses the 160*160 sub-dataset comprising 13124 images of the Abnormal class, denoting Positive results for Gastric Cancer (Cancer Patient), and 20160 images of the Normal class, denoting Negative results for Gastric Cancer (Normal Patient). The detailed summary of the dataset used for the experiments is presented in **Table 2**.

**Table 2:** Summary of GasHisSDB dataset.

Parameter	Value
Dataset used	GasHisSDB (160 × 160 subset)
Staining Method	Hematoxylin and Eosin (H&E)
Magnification	20×
Microscopes Used	Nikon (Japan) and Olympus (Japan)
Cancerous Region Coverage area	50%
Acquisition Software	NewUsbCamera
Data Augmentation	Random rotation and Dataset scrambling
Total Number of Images	33284
Count of Abnormal Images in 160 * 160 subset	13124
Count of Normal Images in 160 * 160 subset	20160
Class Labels	2 Labels (Normal, Abnormal)

## 5 Result analysis

A varied set of experiments is carried out to evaluate the working of the proposed model. The performance of the proposed fusion model (VGG16, RESNET50, and MobileNetv2) was systematically assessed using the GasHisSDB dataset. The results of experiments demonstrate that the fusion approach significantly enhances classification accuracy compared to individual models. Different DL models are initially applied to the GasHisSDB dataset to measure their performance in classifying gastric cancer images. Different CNN-based architectures, including VGG16, RESNET50, MobileNetv2, InceptionV3, DenseNet, and EfficientNet, are implemented and tested based on various evaluation metrics. After a comprehensive performance analysis, the top three performing models—VGG16, RESNET50, and MobileNetv2—are selected for fusion. These architectures are chosen due to their ability to capture diverse spatial hierarchies, deep feature representations, and computational efficiency. LIME provides interpretability to the fused model’s predictions. LIME creates visual explanations by emphasizing the regions of an image that significantly influence the classification choice. LIME finds those crucial regions that the fused model concentrates on when differentiating between cancerous and non-cancerous tissues by varying input photos and examining the model’s reaction. This explainability mechanism is essential in medical diagnostics since it guarantees openness and fosters confidence among medical practitioners. The reliability of the suggested fusion strategy in gastric cancer detection is further supported by the highlighted areas in LIME-based representations matching clinically significant locations.

The first experiment applies different deep learning architectures to the GasHisSDB dataset to assess its effectiveness in gastric cancer detection. Various deep learning models, namely CNN, AlexNet, GoogleNet, RESNET50, VGG16, InceptionV3, EfficientNetB0, MobileNetv2, and Xception, are implemented, and their performance is measured. These models are selected due to their widespread use and performance in image classification tasks, especially in medical imaging. Each model is finetuned and optimized to identify significant features from histopathology pictures and enable precise classification. The whole dataset is split into two parts, where 75% of the data is supplied for training the model, and the remaining 25% of the data is used as testing data for evaluating the model’s performance. This partitioning method is selected to maintain an independent set for reliable evaluation and supply enough training data for model optimization. With 50 epochs in the training phase, the models were able to pick up on intricate patterns in the dataset gradually. To balance learning stability and computational efficiency, a batch size of 32 was used. The experimental results are summarized in **Table 3**. The experimental results in **Table 3** demonstrate superior VGG16, RESNET50, and MobileNetV2 performances compared to other models. The consistency of these three models in detecting gastric cancer indicates their robustness. Because of their remarkable performance, these three models are then considered for fusion in the following experiment to improve the results in comparison to the performance of the individual models.

**Table 3:** Performance results of different DL models on the GasHisSDB dataset.

Model	Accuracy	Precision	Recall	F1-Score
CNN	71.1%	71.8%	71.1%	71.2%
AlexNet	78.9%	79.2%	78.9%	79.0%
GoogLeNet	80.4%	80.6%	80.4%	80.5%
InceptionV3	82.7%	83.0%	82.7%	82.8%
EfficientNetB0	84.3%	84.7%	84.3%	84.5%
Xception	83.5%	83.8%	83.5%	83.6%
**VGG16**	**91.1%**	**92.5%**	**92.7%**	**92.6%**
**RESNET50**	**88.3%**	**93.5%**	**86.7%**	**90.0%**
**MobileNetv2**	**89.8%**	**93.4%**	**89.5%**	**91.4%**

VGG16. RESNET50 and MobileNetV2 present highest accuracy.

Based on the performance of the above experiment, three different models, VGG16 (91% accuracy), RESNET50 (88% accuracy), and MobileNetV2 (89% accuracy), reported the highest accuracy in comparison to others. Hence, these models are concatenated to form a fusion model. The same split criteria of 75:25 is used for the dataset. The fused model was trained using the Adam optimizer for different iterations (epochs), including 10, 20, 35, 50, 70, and 100. After 50 epochs, the validation losses increased, resulting in the model’s over-fitting. Thus, the fused model is trained for 50 epochs involving a batch size of 32. The fusion approach demonstrated superior classification accuracy by leveraging the complementary feature extraction capabilities of RESNET50, VGG16, and MobileNetV2. The fused model recorded an accuracy of 98%. The summary of the results of the fusion model is presented in **Table 4**.

**Table 4:** Performance results of different modalities on the GasHisSDB dataset.

Model	Accuracy	Precision	Recall	F1 Score
VGG16	91.1%	92.5%	92.7%	92.6%
RESNET50	88.3%	93.5%	86.7%	90.0%
MobileNetv2	89.8%	93.4%	89.5%	91.4%
**FUSION (VGG16, RESNET50, MobileNetV2)**	**97.81%**	**98.39%**	**98.00%**	**98.19%**

Fusion Model presents higher accuracy compared to Individual Models.

**Figure 3:**
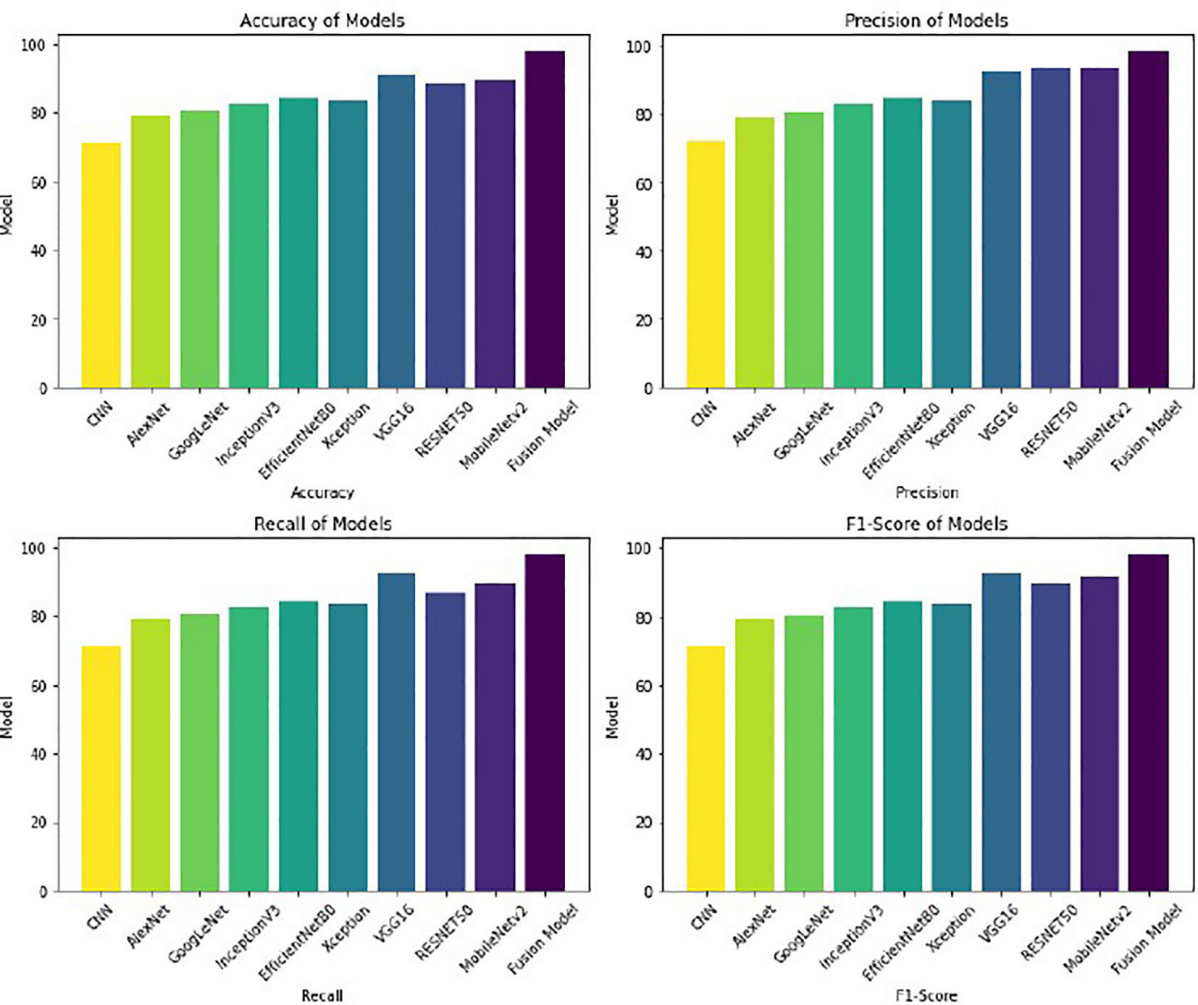
Performance of different models of gastric cancer detection.

The results are better visualized as shown in **Figure 3**, where each model’s performance metrics—Accuracy, Precision, Recall, and F1-Score—are clearly depicted in bar chart form. The Fusion Model outperforms the individual models in all metrics, demonstrating enhancement in overall performance. This graphical visualization presents a clear comparison of various models and the effectiveness of the Fusion model with respect to other models.

**Figure 4:**
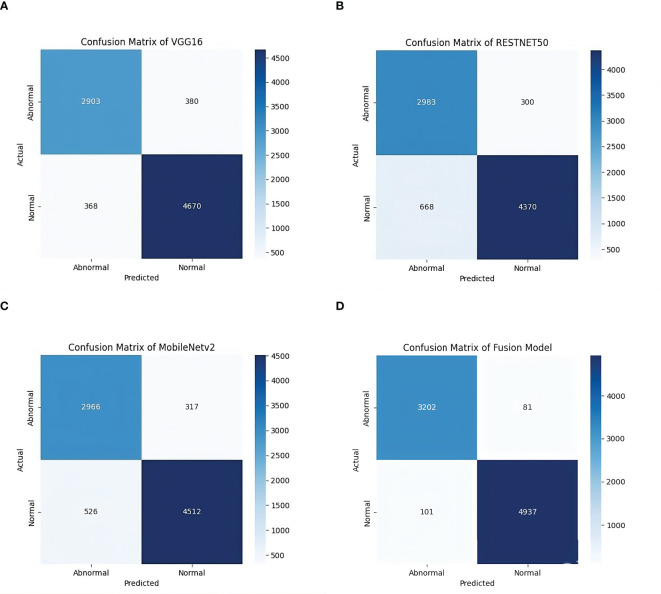
Confusion matrices of individual models and the fused model. **(a)** Confusion Matrix of VGG16 Model on GasHisSDB dataset. **(b)** Confusion Matrix of RESNET50 Model on GasHisSDB dataset. **(c)** Confusion Matrix of MobileNetV2 Model on GasHisSDB dataset. **(d)** Confusion Matrix of Fusion (VGG16 +RESNET50 + MobileNetv2) Model on GasHisSDB dataset.

With an accuracy of around 98%, the fused model outperforms the individual models and records approximately 7% higher accuracy compared to VGG16, the best-performing standalone model. The various feature extraction skills of the separate models, which support one another during the fusion process, are responsible for the performance boost. The confusion matrix of the fusion model compared to the confusion matrices of the best-performing individual models is presented in **Figure 4**. The confusion matrices show how well each model performs in classification by showing how many observations are correctly and erroneously predicted. VGG16, RESNET50, and MobileNetV2 demonstrate strong classification abilities. However, some misclassifications occur. By combining the advantages of three separate architectures, the fusion model exhibits better prediction performance, making more accurate classifications and fewer incorrect ones. This demonstrates how well the model fusion strategy increases overall stomach cancer detection accuracy and dependability.

To improve the fused model’s interpretability, we visualized the key areas inside the image affecting classification choices using LIME (Local Interpretable Model-agnostic Explanations). After creating altered versions of an input image and assessing the model’s reaction, LIME builds a locally interpretable surrogate model that emphasizes the most significant areas. Multiple images of each class, normal and abnormal images, were chosen for this investigation. LIME is applied to examine how the fused model distinguishes between cancer-infected and non-cancerous areas. The regions of the histopathological pictures that are most important to the model’s categorization are highlighted in the LIME visualizations produced. According to the findings, the fused model mainly concentrates on critical cellular components linked to stomach cancer, guaranteeing that predictions are based on biologically significant characteristics.

**Figure 5:**
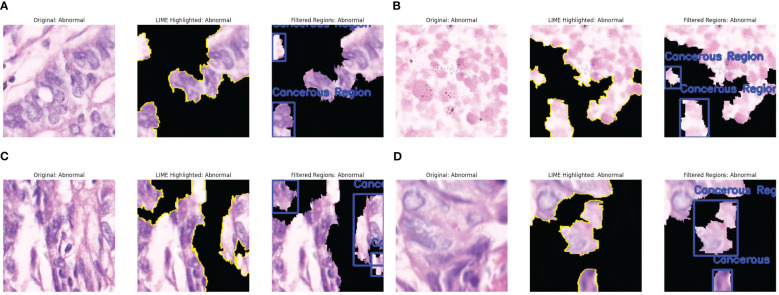
LIME explanations for Cancerous Images. **(a)** Cancer Abnormality observed: Case 1. **(b)** Cancer Abnormality observed: Case 2. **(c)** Cancer Abnormality observed: Case 3. **(d)** Cancer Abnormality observed: Case 4.

**Figure 5** shows the results of the LIME technique applied to the Cancerous Images. Every resultant image consists of three parts: the original image, the LIME highlighted results, and the image having annotations describing the malignant or benign regions. The black-shaded portions in the pictures represent less importance of those pixels in the model’s prediction. **Figure 5a** shows that LIME highlights the five critical regions facilitating cancer prediction. The LIME technique presents the images in which the highlighted sections are represented with yellow lines, denoting the critical areas that contribute highly to the model’s decision. This highlighted section shows the presence of irregular cell structures and malignant lesions. The third part of the image presents the abnormal cell structures by annotating them with blue boxes. Similarly, **Figure 5b** shows the widespread distribution of the regions and detects two regions comprising densely packed cell clusters, marking them with the abnormal annotation with blue boxes. More structures and aligned results are observed in **Figure 5c**, showing four different important regions from the image identified by LIME, out of which three areas consist of irregularly shaped and elongated structures representing densely packed cells, indicating a case of abnormality. Similarly, **Figure 5d** identifies the three significant regions, of which two areas resembled abnormalities due to cancer lesions. The LIME explanation effectively identifies the most crucial regions with malignancies in the model’s classification.

**Figure 6:**
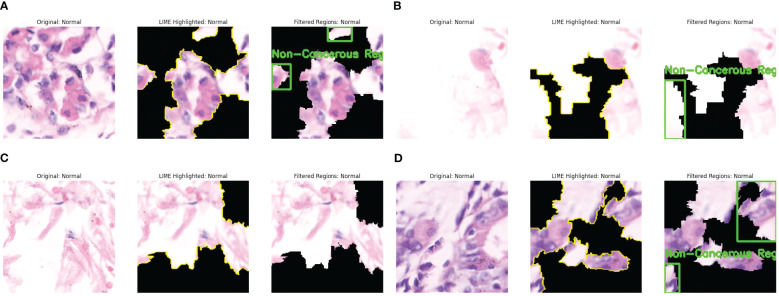
LIME explanations for non-cancerous (normal) images. **(a)** Cancer Abnormality not observed: Case 1. **(b)** Cancer Abnormality not observed: Case 2. **(c)** Cancer Abnormality not observed: Case 3. **(d)** Cancer Abnormality not observed: Case 4.

Similarly, the interpretation of some predictions as Non-cancerous by LIME is presented in **Figure 6**. The significant regions are identified in the original Image. They are highlighted in yellow, and the non-important areas are represented by black, as shown in **Figure 6a**. The cell arrangement in the highlighted section is well-structured, and no irregular cell clustering is observed; hence, the Image is annotated as Non-Cancerous in green boxes. **Figure 6b** shows fewer regions resembling well arranged and organized cell tissues and the absence of abnormal nuclei formation. The highlighted areas present the uniform distribution of the cells without any abnormality. This Image is thus annotated as Non-Cancerous, and green boxes annotate the areas in the last part of the Image. **Figure 6c** shows the absence of dark stains or irregular cell formations, presenting the well-distributed cell structure. A large part of the Image is essential for model decision, represented by LIME with yellow lines. This region shows the absence of irregularities and densely packed cell structures, resulting in the prediction of a non-cancerous class. **Figure 6d** shows the diverse regions contributing to the model’s decision. These regions have evenly distributed cells and have no presence of excessive clustering of cell nuclei. Thus, these regions are annotated as non-cancerous and are highlighted in green boxes. The findings of LIME properly resemble the absence of malignant cells and present explanations about the model’s prediction as Non-Cancerous.

### 5.1 Comparative analysis

We compare our model’s performance with current methodologies and approaches for identifying gastric cancer. Researchers have created many methods and models to increase diagnostic accuracy, using deep learning architectures, machine learning models, and different imaging modalities. To provide an equitable and significant comparison, we mainly concentrate on histological image analysis methods in this investigation. **Table 5** compares our approach with these approaches.

**Table 5:** Comparative analysis.

Reference	Architecture	Accuracy	Explainability (Yes or No)	Explainability Method
(53)	EfficientB0 + DenseNet-201	95%	No	NA
(54)	Deep Belief Network	96%	No	NA
(51)	VGG16	96%	No	NA
(55)	EfficientNetV2B0-CatBoost	93.9%	Yes	GradCAM
(56)	Enhanced EMD Convolutional Neural Network (EECNN)	94.5%	No	NA
(57)	ResNet with Involution operations	91%	No	NA
(46)	DenseNet-201	88.75%	Yes	LIME
(23)	Ensembled Approach (ELM, PD-CNN)	87.75%	Yes	SHAP and Grad-CAM
(58)	Dual-Resolution Attention Capsule Network (DRA-CN)	97.50%	No	NA
(59)	RESNET50	96.09%	No	NA
Proposed Approach	(VGG16 + RESNET50 + MobileNetV2)	97.81%	Yes	LIME

The research in (53) presented the fusion approach, fusing EfficientNetB0 and DenseNet-201 for cancer detection. This approach combines handcrafted and deep features to enhance the classification performance. This approach recorded an accuracy of 95% after applying the cross-magnitude generalizations, and no explanation techniques were used. In (54), the researchers employ the Deep Belief Network (DBN) for predicting cancer. This approach records an accuracy of 96% with the absence of any explanations. This approach uses the statistical two-tailed test to test the performance of DBN. This approach samples the data into 40 images per sample, raising concerns about generalization. Hu et al. in (51) presented using the VGG16 model for detecting gastric cancer. This experiment was run for 100 epochs, resulting in higher computational times and increased risk of over-fitting. This approach recorded an accuracy of 96%, and no explanations about the decisions are presented. The authors in (55) present the hybrid approach, combining a DL and boosting models for detecting cancer. This approach employs the EfficientNetV2B0 for extracting features and employs CatBoost for classification purposes. This approach records an accuracy of 93.9%. It presents the GradCAM visualizations, explaining the model’s decisions in heat maps—the fusion of EfficientNetV2B0 and CatBoost results in higher computational times.

The study in (56) presents the Enhanced EMD-CNN (EEMDCNN) model for automated detection of gastric cancer. This approach uses the empirical mode decomposition (EMD) to extract the intrinsic features by adding multi-resolution deconvolutional filters. This approach is susceptible to noise and varying intensities of inputs and records an accuracy of 94.5%, resulting in higher computational times due to additional convolutional layers. No explanations about the decisions are presented. The researchers in (57) employ the use of ResInvolution, a combination of ResNet and Involution operations, to adjust the spatial weights of the features. This approach records an accuracy of 91% and is implemented in a Federated Environment, achieving data privacy. In (46), the authors presented a method using the DenseNet-201 model, a pre-trained CNN variant. This approach implements the LIME technique for interpreting results and achieves an accuracy of 88.75%. The researchers in (23) present the ensemble learning model comprising a parallel depth-wise separable CNN, Extreme Learning Machine (ELM), and L1 Regularized ELM. This model records an accuracy of 87.75%, and the model’s decisions are explained with the help of SHAP, denoting the essential features and representing them with Grad-Cam. The study in (58) proposes the Dual-Resolution Attention Capsule Network (DRA-CN) to improve the classification of histopathological images. This approach employs the attention-based approach to enhance categorical learning and presents dynamic routing optimization that helps better generalize the model. This technique presents an accuracy of 97.5% with any interpretability features or without implementing any XAI technique. Hu. et al. in (59) employed the RESNET50 model on the histopathological images for detecting gastric cancer. This approach recorded an accuracy of 96.09% in the absence of interpretations of results. This approach showed enhanced performance in contrast to the traditional ML models. The need for an XAI technique for ensuring transparency and trust is missing in this approach. The detailed summary of the comparative analysis is presented in **Table 5**.

Our proposed fusion model, fusing VGG16, RESNET50, and MobileNetV2, recorded an accuracy of 97.81%, surpassing various state-of-the-art methodologies for detecting GC using Histopathological images in the GasHisSDB dataset. Several approaches presented in the comparative analysis lack the employment of the XAI technique, which ensures trust in the model’s decisions. Some methods have employed XAI techniques for result interpretation, but the accuracy achieved by those models is very low compared to our approach. Integration of different architectures facilitates robust feature extraction with enhanced classification accuracy. Our proposed model extracts the high-level global features and the fine-tuned local features that enhance performance. With the addition of LIME, the clinicians receive the highlighted sections of the Images that help them validate the model’s predictions. This allows them to discover the facts behind the model’s predictions and creates a feeling of trust for them toward AI-driven cancer detection systems.

## 6 Conclusion

The proposed fusion methodology, fusing VGG16, RESNET50, and MobileNetV2, for analyzing histopathological images for gastric cancer detection delivers promising results and presents an accuracy of 97.81% which is higher compared to the individual models- VGG16 (91%), RESNET50(88%), and MobileNetV2 (89%). The proposed methods present superior results in contrast to state-of-the-art methods for GC detection. The proposed model leverages the strength of the individual models, extracting the high-level and the low-level features. With the incorporation of LIME, the model achieves the trust of medical professionals in cancer diagnostics as LIME presents the highlighted sections of the Images contributing towards the model’s decision. Our method offers a well-balanced solution that promotes precision and transparency, in contrast to previous models that are either less accurate or lack interpretability. Explainability add-ons are handy in practical healthcare cases because one needs to understand how the model arrived at certain decisions.

However, some challenges still exist in handling the training methods and the imaging conditions, which cause problems in modeling the generalization across different datasets. We plan to include more generalization features in the Future, making our model acceptable to diverse datasets. In the Future, we plan to employ more advanced feature selection techniques and optimize our model to reduce computational time while preserving accuracy and performance. In the Future, we also plan to implement the federated learning approach to ensure privacy and explore self-supervised and unsupervised learning techniques for more accurate and comprehensive AI-driven cancer detection systems.

## Data Availability

The raw data supporting the conclusions of this article will be made available by the authors, without undue reservation.

## References

[1] Pasechnikov V Chukov S Fedorov E Kikuste I Leja M. Gastric cancer: prevention, screening and early diagnosis. World J gastroenterol: WJG. (2014) 20:13842. doi: 10.3748/wjg.v20.i38.13842 25320521 PMC4194567

[2] Xia JY Aadam AA. Advances in screening and detection of gastric cancer. J Surg Oncol. (2022) 125:1104–9. doi: 10.1002/jso.v125.7 PMC9322671 35481909

[3] Necula L Matei L Dragu D Neagu AI Mambet C Nedeianu S. Recent advances in gastric cancer early diagnosis. World J gastroenterol. (2019) 25:2029. doi: 10.3748/wjg.v25.i17.2029 31114131 PMC6506585

[4] Ma S Zhou M Xu Y Gu X Zou M Abudushalamu G. Clinical application and detection techniques of liquid biopsy in gastric cancer. Mol Cancer. (2023) 22 7. doi: 10.1186/s12943-023-01715-z 36627698 PMC9832643

[5] Dohi O Seya M Iwai N Ochiai T Yumoto J Mukai H. Endoscopic detection and diagnosis of gastric cancer using image-enhanced endoscopy: A systematic review and meta-analysis. DEN Open. (2025) 5:e418. doi: 10.1002/deo2.v5.1 39144408 PMC11322228

[6] Chen Y Wang B Zhao Y Shao X Wang M Ma F. Metabolomic machine learning predictor for diagnosis and prognosis of gastric cancer. Nat Commun. (2024) 15:1657. doi: 10.1038/s41467-024-46043-y 38395893 PMC10891053

[7] Öztürk Şaban Özkaya U. Residual lstm layered cnn for classification of gastrointestinal tract diseases. J Biomed Inf. (2021) 113:103638. doi: 10.1016/j.jbi.2020.103638 33271341

[8] Xie K Peng J. Deep learning-based gastric cancer diagnosis and clinical management. J Radiat Res Appl Sci. (2023) 16:100602. doi: 10.1016/j.jrras.2023.100602

[9] Zhang S Yuan Z Zhou X Wang H Chen Bo Wang Y. Venet: Variational energy network for gland segmentation of pathological images and early gastric cancer diagnosis of whole slide images. Comput Methods Programs Biomed. (2024) 250:108178. doi: 10.1016/j.cmpb.2024.108178 38652995

[10] Ding S Hu S Li X Zhang Y Wu DD. Leveraging multimodal semantic fusion for gastric cancer screening via hierarchical attention mechanism. IEEE Trans Systems Man Cybernetics: Syst. (2021) 52:4286–99. doi: 10.1109/TSMC.2021.3096974

[11] Zheng X Wang R Zhang X Sun Y Zhang H Zhao Z. A deep learning model and human-machine fusion for prediction of ebv-associated gastric cancer from histopathology. Nat Commun. (2022) 13:2790. doi: 10.1038/s41467-022-30459-5 35589792 PMC9120175

[12] Gurcan F. Enhancing breast cancer prediction through stacking ensemble and deep learning integration. PeerJ Comput Sci. (2025) 11:e2461. doi: 10.7717/peerj-cs.2461 PMC11888877 40062255

[13] Mathivanan SK Francis D Srinivasan S Khatavkar V Karthikeyan P Shah MA. Enhancing cervical cancer detection and robust classification through a fusion of deep learning models. Sci Rep. (2024) 14:10812. doi: 10.1038/s41598-024-61063-w 38734714 PMC11088661

[14] Lucky Lhaura Van FC Khairul Anam M Bukhori S Mahamad AK Saon S La Volla Nyoto R. The development of stacking techniques in machine learning for breast cancer detection. J Appl Data Sci. (2025) 6:71–85. doi: 10.47738/jads.v6i1.416

[15] Wafa AA Essa RM Abohany AA Abdelkader HE. Integrating deep learning for accurate gastrointestinal cancer classification: a comprehensive analysis of msi and mss patterns using histopathology data. Neural Computing Appl. (2024) 36:1–33. doi: 10.1007/s00521-024-10287-y

[16] Duan J Xiong J Li Y Ding W. Deep learning based multimodal biomedical data fusion: An overview and comparative review. Inf Fusion. (2024) 112:102536. doi: 10.1016/j.inffus.2024.102536

[17] Ni W Wang T Wu Yu Liu X Li Z Yang R. Multi-task deep learning model for quantitative volatile organic compounds analysis by feature fusion of electronic nose sensing. Sensors Actuators B: Chem. (2024) 417:136206. doi: 10.1016/j.snb.2024.136206

[18] Raju ASN Jayavel K Rajalakshmi T Rajababu M. Crcfusionaicadx: Integrative cnn-lstm approach for accurate colorectal cancer diagnosis in colonoscopy images. Cogn Comput. (2025) 17:1–37. doi: 10.1007/s12559-024-10357-2

[19] Chempak Kumar A Muhammad Noorul Mubarak D. Evaluation of gastric cancer using explainable ai techniques. In: International Conference on Information and Management Engineering. Hyderabad, India: Springer (2022). p. 87–98.

[20] de Souza LA Jr Mendel R Strasser S Ebigbo A Probst A Messmann H. Convolutional neural networks for the evaluation of cancer in barrett’s esophagus: Explainable ai to lighten up the black-box. Comput Biol Med. (2021) 135:104578. doi: 10.1016/j.compbiomed.2021.104578 34171639

[21] Varanasi LVSKBK Sumathi D Natarajan K. Gastric cancer detection using hybridbased network and shap analysis. In: Explainable Artificial Intelligence for Biomedical Applications. New York, USA: River Publishers (2023). p. 1–16.

[22] Longo L Brcic M Cabitza F Choi J Confalonieri R Ser JD. Explainable artificial intelligence (xai) 2.0: A manifesto of open challenges and interdisciplinary research directions. Inf Fusion. (2024) 106:102301. doi: 10.1016/j.inffus.2024.102301

[23] Ahamed MdF Nahiduzzaman Md Islam MdR Naznine M Ayari MA Khandakar A. Detection of various gastrointestinal tract diseases through a deep learning method with ensemble elm and explainable ai. Expert Syst With Appl. (2024) 256:124908. doi: 10.1016/j.eswa.2024.124908

[24] Binzagr F. Explainable ai-driven model for gastrointestinal cancer classification. Front Med. (2024) 11:1349373. doi: 10.3389/fmed.2024.1349373 PMC11056557 38686367

[25] Vimbi V Shaffi N Mahmud M. Interpreting artificial intelligence models: a systematic review on the application of lime and shap in alzheimer’s disease detection. Brain Inf. (2024) 11:10. doi: 10.1186/s40708-024-00222-1 PMC10997568 38578524

[26] Salih AM Raisi-Estabragh Z Galazzo IB Radeva P Petersen SE Lekadir K. A perspective on explainable artificial intelligence methods: Shap and lime. Advanced Intelligent Syst. (2025) 7:2400304. doi: 10.1002/aisy.202400304

[27] Leersum CMv Maathuis C. Human centred explainable ai decision-making in healthcare. J Responsible Technol. (2025) 21:100108. doi: 10.1016/j.jrt.2025.100108

[28] Hallissey MT Allum WH Jewkes AJ Ellis DJ Fielding JW. Early detection of gastric cancer. Br Med J. (1990) 301:513–5. doi: 10.1136/bmj.301.6751.513 PMC1663798 2207416

[29] Niu P-H Zhao L-L Wu H-L. Dong-Bing Zhao, and Ying-Tai Chen. Artificial intelligence in gastric cancer: Application and future perspectives. World J gastroenterol. (2020) 26:5408. doi: 10.3748/wjg.v26.i36.5408 33024393 PMC7520602

[30] Jamil D Palaniappan S Lokman A Naseem M Zia SS. Diagnosis of gastric cancer using machine learning techniques in healthcare sector: a survey. Informatica. (2022) 45:2022. doi: 10.31449/inf.v45i7.3633

[31] Li Y Li X Xie X Shen L. (2018). Deep learning based gastric cancer identification, in: 2018 IEEE 15th international symposium on biomedical imaging (ISBI 2018). pp. 182–5. Washington DC USA : IEEE.

[32] Patel AD Jhaveri RH Patel AD Shah KA Shah J. Enhanced aiot multi-modal fusion for human activity recognition in ambient assisted living environment. Software: Pract Experience. (2024) 2024:731–47. doi: 10.1002/spe.3394

[33] Renjith VR Judith JE. (2023). Explainable artificial intelligence for gastrointestinal cancer using cnn-a review, in: AIP Conference Proceedings, Vol. 2904. Kollam, Kerala: AIP Publishing.

[34] Zhao Y Hu Bo Wang Y Yin X Jiang Y Zhu X. Identification of gastric cancer with convolutional neural networks: a systematic review. Multimedia Tools Appl. (2022) 81:11717–36. doi: 10.1007/s11042-022-12258-8 PMC8856868 35221775

[35] Ikenoyama Y Hirasawa T Ishioka M Namikawa K Yoshimizu S Horiuchi Y. Detecting early gastric cancer: Comparison between the diagnostic ability of convolutional neural networks and endoscopists. Digestive Endoscopy. (2021) 33:141–50. doi: 10.1111/den.13688 PMC7818187 32282110

[36] Li L Chen Y Shen Z Zhang X Sang J Ding Y. Convolutional neural network for the diagnosis of early gastric cancer based on magnifying narrow band imaging. Gastric Cancer. (2020) 23:126–32. doi: 10.1007/s10120-019-00992-2 PMC6942561 31332619

[37] Zhu Y Wang Q-C Xu M-D Zhang Z Cheng J Zhong Y-S. Application of convolutional neural network in the diagnosis of the invasion depth of gastric cancer based on conventional endoscopy. Gastrointestinal endoscopy. (2019) 89:806–15. doi: 10.1016/j.gie.2018.11.011 30452913

[38] Hirasawa T Aoyama K Tanimoto T Ishihara S Shichijo S Ozawa T. Application of artificial intelligence using a convolutional neural network for detecting gastric cancer in endoscopic images. Gastric Cancer. (2018) 21:653–60. doi: 10.1007/s10120-018-0793-2 29335825

[39] Mudavadkar GR Deng Mo Al-Heejawi SMA Arora IH Breggia A Ahmad B. Gastric cancer detection with ensemble learning on digital pathology: Use case of gastric cancer on gashissdb dataset. Diagnostics. (2024) 14:1746. doi: 10.3390/diagnostics14161746 39202233 PMC11354078

[40] Mohammad F Al-Razgan M. Deep feature fusion and optimization-based approach for stomach disease classification. Sensors. (2022) 22:2801. doi: 10.3390/s22072801 35408415 PMC9003289

[41] Mao S Liu J. Mulitdeepsurv: survival analysis of gastric cancer based on deep learning multimodal fusion models. Biomed Optics Express. (2024) 16:126–41. doi: 10.1364/BOE.541570 PMC11729289 39816158

[42] Zhang H Lu M Chen W Ao F Chai Yi. (2024). An early gastric cancer detection method based on detr multi-scale feature fusion, in: 2024 43rd Chinese Control Conference (CCC). pp. 7304–9. Kunming, China: IEEE.

[43] Shi Xu Wang L Li Yu Wu J Huang H. Gcldnet: Gastric cancer lesion detection network combining level feature aggregation and attention feature fusion. Front Oncol. (2022) 12:901475. doi: 10.3389/fonc.2022.901475 36106104 PMC9464831

[44] Haq EUl Yong Q Yuan Z Jianjun H Haq RUl Qin X. Accurate multiclassification and segmentation of gastric cancer based on a hybrid cascaded deep learning model with a vision transformer from endoscopic images. Inf Sci. (2024) 670:120568. doi: 10.1016/j.ins.2024.120568

[45] Auzine MM Khan MH-M Baichoo S Sahib NG Bissoonauth-Daiboo P Gao X. Development of an ensemble cnn model with explainable ai for the classification of gastrointestinal cancer. PloS One. (2024) 19:e0305628. doi: 10.1371/journal.pone.0305628 38917159 PMC11198752

[46] Shaw P Sankaranarayanan S Lorenz P. (202). Early esophageal Malignancy detection using deep transfer learning and explainable ai, in: 2022 6th International Conference on Communication and Information Systems (ICCIS). pp. 129–35. Chongqing, China: IEEE.

[47] Afeni BO Adetunji PA Yakub IO Adetunji PA. Interpretable machine learning approach for early cancer detection. Int J Sci Res Arch. (2024) 13. doi: 10.30574/ijsra.2024.13.2.2586

[48] Theckedath D Sedamkar RR. Detecting affect states using vgg16, resnet50 and se-resnet50 networks. SN Comput Sci. (2020) 1:79. doi: 10.1007/s42979-020-0114-9

[49] Elpeltagy M Sallam H. Automatic prediction of covid- 19 from chest images using modified resnet50. Multimedia Tools Appl. (2021) 80:26451–63. doi: 10.1007/s11042-021-10783-6 PMC8095476 33967592

[50] Gulzar Y. Fruit image classification model based on mobilenetv2 with deep transfer learning technique. Sustainability. (2023) 15:1906. doi: 10.3390/su15031906

[51] Hu W Li C Li X Rahaman MdM Ma J Zhang Y. Gashissdb: A new gastric histopathology image dataset for computer aided diagnosis of gastric cancer. Comput Biol Med. (2022) 142:105207. doi: 10.1016/j.compbiomed.2021.105207 35016101

[52] Yong MP Hum YC Lai KW Lee YL Goh C-H Yap W-S. Histopathological gastric cancer detection on gashissdb dataset using deep ensemble learning. Diagnostics. (2023) 13:1793. doi: 10.3390/diagnostics13101793 37238277 PMC10217020

[53] Loddo A Usai M Ruberto CDi. Gastric cancer image classification: A comparative analysis and feature fusion strategies. J Imaging. (2024) 10 195. doi: 10.3390/jimaging10080195 39194984 PMC11355805

[54] Koushik TS Kothandapani S Sheela JJJ. (2024). Efficient gastric cancer detection using deep belief network compared over support vector machine with improved accuracy, in: 2024 9th International Conference on Applying New Technology in Green Buildings (ATiGB). pp. 491–6. Danang, Vietnam: IEEE.

[55] Khayatian D Maleki A Nasiri H Dorrigiv M. Histopathology image analysis for gastric cancer detection: a hybrid deep learning and catboost approach. Multimedia Tools Appl. (2024), 1–27. doi: 10.1007/s11042-024-19816-2

[56] Rasal T Veerakumar T Subudhi BN Esakkirajan S. Segmentation of gastric cancer from microscopic biopsy images using deep learning approach. Biomed Signal Process Control. (2023) 86:105250. doi: 10.1016/j.bspc.2023.105250

[57] Dipto SM. *ResInvolution: an involution-ResNet fused global* sp*atial relation leveraging model for histopathological image analysis under federated learning environment* . Bangladesh: Brac University (2024).

[58] Tursun P Li S Li M Lv X Chen C Chen C. (2024). Dra-cn: A novel dual-resolution attention capsule network for histopathology image classification, in: Chinese Conference on Pattern Recognition and Computer Vision (PRCV). pp. 209–22. Urumqi, China: Springer.

[59] Hu W Chen H Liu W Li X Sun H Huang X. A comparative study of gastric histopathology sub-size image classification: From linear regression to visual transformer. Front Med. (2022) 9:1072109. doi: 10.3389/fmed.2022.1072109 PMC9767945 36569152

